# Non-viral-mediated gene transfer of OX40 ligand for tumor immunotherapy

**DOI:** 10.3389/fimmu.2024.1410564

**Published:** 2024-06-26

**Authors:** Olga A. Rakitina, Alexey I. Kuzmich, Olga A. Bezborodova, Sofia A. Kondratieva, Victor V. Pleshkan, Marina V. Zinovyeva, Dmitry A. Didych, Aleksandr V. Sass, Eugene V. Snezhkov, Maria B. Kostina, Maksim O. Koksharov, Irina V. Alekseenko

**Affiliations:** ^1^ Group of Gene Immuno-Oncotherapy, Department of Genomics and Postgenomic Technologies, Shemyakin-Ovchinnikov Institute of Bioorganic Chemistry of the Russian Academy of Sciences, Moscow, Russia; ^2^ Laboratory of Human Gene Structure and Functions, Department of Genomics and Postgenomic Technologies, Shemyakin-Ovchinnikov Institute of Bioorganic Chemistry of the Russian Academy of Sciences, Moscow, Russia; ^3^ Stagen LLC, Moscow, Russia

**Keywords:** gene-immune therapy, OX40L, non-viral gene therapy, gene therapy, cancer, polyplexes, OX40 agonist

## Abstract

**Background:**

Immune checkpoint blockade (ICB) is rapidly becoming a standard of care in the treatment of many cancer types. However, the subset of patients who respond to this type of therapy is limited. Another way to promote antitumoral immunity is the use of immunostimulatory molecules, such as cytokines or T cell co-stimulators. The systemic administration of immunotherapeutics leads to significant immune-related adverse events (irAEs), therefore, the localized antitumoral action is needed. One way to achieve this is intratumoral non-viral gene-immune therapy, which allows for prolonged and localized gene expression, and multiple drug administration. In this study, we combined the previously described non-viral gene delivery system, PEG-PEI-TAT copolymer, PPT, with murine OX40L-encoding plasmid DNA.

**Methods:**

The resulting OX40L/PPT nanoparticles were characterized via gel mobility assay, dynamic light scattering analysis and *in vitro* transfection efficiency evaluation. The antitumoral efficacy of intratumorally (i.t.) administered nanoparticles was estimated using subcutaneously (s.c.) implanted CT26 (colon cancer), B16F0 (melanoma) and 4T1 (breast cancer) tumor models. The dynamics of stromal immune cell populations was analyzed using flow cytometry. Weight loss and cachexia were used as irAE indicators. The effect of combination of i.t. OX40L/PPT with intraperitoneal PD-1 ICB was estimated in s.c. CT26 tumor model.

**Results:**

The obtained OX40L/PPT nanoparticles had properties applicable for cell transfection and provided OX40L protein expression *in vitro* in all three investigated cancer models. We observed that OX40L/PPT treatment successfully inhibited tumor growth in B16F0 and CT26 tumor models and showed a tendency to inhibit 4T1 tumor growth. In B16F0 tumor model, OX40L/PPT treatment led to the increase in antitumoral effector NK and T killer cells and to the decrease in pro-tumoral myeloid cells populations within tumor stroma. No irAE signs were observed in all 3 tumor models, which indicates good treatment tolerability in mice. Combining OX40L/PPT with PD-1 ICB significantly improved treatment efficacy in the CT26 subcutaneous colon cancer model, providing protective immunity against CT26 colon cancer cells.

**Conclusion:**

Overall, the anti-tumor efficacy observed with OX40L non-viral gene therapy, whether administered alone or in combination with ICB, highlights its potential to revolutionize cancer gene therapy, thus paving the way for unprecedented advancements in the cancer therapy field.

## 1 Introduction

Immune checkpoint blockade (ICB) has dramatically changed the clinical paradigm of cancer treatment (1). The currently approved (2) systemically administered checkpoint inhibitors have improved metastatic disease survival by targeting the T cell co-inhibitory pathways of CTLA-4 (cytotoxic T-lymphocyte associated protein 4; ipilimumab (3, 4) tremelimumab (5, 6)), PD-1/PD-L1 (programmed cell death protein 1/programmed death-ligand 1; nivolumab, pembrolizumab, cemiplimab, atezolizumab, avelumab, durvalumab (4), dostarlimab (7)) and LAG-3 (lymphocyte-activation gene 3; relatlimab (8)). Besides, therapies targeting other immune checkpoints are currently developed (extensively reviewed by Marin-Acevedo et al. (4) and Meybodi et al. (9)). The currently approved antibodies have provided durable responses in some patient cohorts, but many tumors are ICB-resistant. The ability of different tumors to respond to ICB in the case of anti-PD-1/PD-L1 checkpoint immunotherapy is largely determined by the inflammatory state of the tumor microenvironment (10) – while more-inflamed “hot” tumors respond strongly, even in advanced cases, the less-inflamed “cold” tumors respond poorly, or do not respond at all (11). One of the approaches to increase ICB efficiency is to attempt to “heat up” the “cold” tumors via combining ICB with immunostimulation. Such immunostimulation can be achieved, in example, using immunostimulatory cytokines (12, 13) and T cell co-stimulator ligands (14, 15). One of the well-known T cell co-stimulators, which is currently actively targeted in various preclinical and clinical trials, is OX40. The targeting strategies vary from using systemically injected OX40 agonists (i.e., anti-OX40 monoclonal antibodies) to intratumorally injected gene therapies, i.e., lipid nanoparticle-encapsulated OX40 ligand (OX40L)-encoding mRNA or OX40L-expressing oncolytic adenoviruses (15). While being more convenient, the systemically administered therapies have several significant disadvantages, including the production cost, the off-target effects and systemic toxicity leading to immune-related adverse events (16) and, in some cases, therapy discontinuation (17). In turn, the locally-administered gene therapies may lack thereof.

Gene therapy approaches can be divided into viral and non-viral, based on the genetic material carrier. Whereas viral approaches are most commonly used due to efficient cell transduction and subsequent protein production, the use of viral-based therapeutics can be limited by immunogenicity, cytotoxicity, potential carcinogenicity and production cost (18, 19). Therefore, the non-viral gene therapy approaches are being actively developed.

We have previously reported data on the antitumor efficacy of nonviral PPT gene delivery system carrying plasmid DNA. The plasmid DNA encoded a suicide gene, herpes virus thymidine kinase, and a granulocyte-macrophage colony stimulating factor (20). This system has proven itself effective and safe in preclinical studies, and is currently undergoing Phase I clinical investigation, NCT05578820 (21).

We supposed that combination of the PPT gene delivery system with OX40L-encoding plasmid DNA will provide substantial antitumor effect without causing serious adverse events. In this study, we combined the previously described gene delivery system with OX40L-encoding plasmid DNA, characterized the resulting OX40L/PPT nanoparticles and evaluated their efficiency in several *in vivo* murine tumor models. We also investigated the effect of OX40L/PPT combination with anti-PD-1 ICB.

## 2 Materials and methods

### 2.1 Materials

Plasmid DNA encoding murine OX40L was previously obtained (22). The cationic copolymer PPT was obtained as described previously (23) using PEI (cat. no. 23966, Polysciences, Warrington, PA), PEG (cat. no. 10314, Quanta BioDesign, Powell, OH) and TAT peptide (GRKKKRRQRC, synthesized by RusBiolink, Moscow, Russia). DMEM/F12 (cat. no. A4192001), RPMI-1640 (cat. no. A4192301), Fetal Bovine Serum (FBS, cat. no. A5256701), Antibiotic-Antimycotic (100×, cat. no. 15240062), Trypsin-EDTA (cat. no. 25300054), TripLE™ Express Enzyme (cat. no. 12605028), and Opti-MEM (cat. no. 31985070) were obtained from Gibco (Carlsbad, CA, USA); Trypan Blue Stain (0.4%, cat. no. T10282), and Lipofectamine^®^ 2000 (cat. no. 11668500) were obtained from Invitrogen, USA. All the oligonucleotide primers were synthesized by Evrogen (Moscow, Russia). All other chemical reagents were obtained commercially as reagent grade products.

### 2.2 Cell lines

We used the following cell lines: B16F0 murine melanoma (obtained from ATCC, CRL-6322™), CT26gfp murine undifferentiated colon carcinoma, that is, CT26.WT, ATCC^®^ CRL-2638™ modified for stable expression of EGFP, which was kindly provided by E.O. Serebrovskaya (24). The 4T1 murine breast cancer (obtained from ATCC, CRL-2539). B16F0 cell line was cultured in DMEM/F12 medium (1:1) supplemented with 10% FBS and antibiotic-antimycotic solution in a 5% CO_2_ incubator at 37°C under 95% humidity. CT26gfp and 4T1 cell lines were cultured in RPMI-1640 medium supplemented with 12.5% FBS and antibiotic-antimycotic solution in a 5% CO_2_ incubator at 37°C under 95% humidity. All the cell lines were subcultured every 3–4 days using trypsin-EDTA. When necessary, the cells were collected with trypsin-EDTA, stained with trypan blue stain, and counted using the Countess II FL Automated Cell Counter (Invitrogen).

### 2.3 Polyplex preparation and characterization

The PPT/pDNA complexes were prepared as described previously (23) in HEPES-glucose buffer (5% glucose, 5мМ HEPES, рН 7,5). Briefly, PPT solution in borate buffer (0.1 M borate, pH 7.5) was vigorously mixed with 1 volume of 4x HEPES-glucose buffer and 2 volumes of 2x plasmid DNA solution (water, 160 ng/μL pDNA) and incubated for 40 min at room temperature (RT, 18–25˚C) before use. The resulting solutions had N/P ratio 0.5, 1, 5, 10, 20 or 30 depending on the initial PPT solution concentration. For *in vitro* transfection efficiency and *in vivo* studies, the N/P=30 was used, the resulting solution contained 12,5mkM PPT, 80 ng/μL plasmid DNA. The PEG/PEI ratio was optimized according to Ulasov et al. (23)

The plasmid DNA charge neutralization was evaluated with agarose gel mobility assay. The PPT/pDNA complexes were prepared using murine OX40L-encoding pDNA (22) at N/P ratios 0.5, 1, 5, 10, 20 and 30 as described above. As a control, PPT-free borate buffer was used instead of PPT solution (N/P=0). The resulting complexes were subjected to agarose gel electrophoresis (1% agarose).

The particle size of the resulting complexes was measured by dynamic light scattering using a Brookhaven 90plus particle size analyzer (Brookhaven Instruments, Holtsville, NY, USA). Measurements were performed in a plastic cuvette at 25°C in ten runs of 20 s duration each and analyzed in MSD (multimodal size distribution) analysis using 90Plus Particle sizing software. Zeta potential of the particles was measured by electrophoretic light scattering using a Brookhaven 90plus particle size analyzer with the BI-PALS module (Brookhaven Instruments, Holtsville, NY, USA) in the same conditions and calculated using PALS Zeta Potential Analyzer software (Ver, 5.78, Brookhaven Instruments, Holtsville, NY, USA).

### 2.4 *In vitro* evaluation of transfection efficiency

The transfection efficiency was evaluated using flow cytometry. For that, СT26gfp, B16F0 and 4T1 cells were plated in 6-well plates (5 × 10^5 cells/well) in full media and incubated in a 5% CO_2_ incubator at 37°C for 24 h. The next day, when the cells reached 40–60% confluence, the transfection complexes containing 12,5 mkM PPT, 80 ng/μL pDNA (N/P=30) were prepared as described above. After the preparation, the complexes were added into fresh full media (2 μg pDNA per well) and the old growth media in the 6-well plates was replaced with the resulting transfection mixture. Transfection with Lipofectamine^®^ 2000 according to manufacturer’s protocol was used as a positive control (2 μg pDNA per well). Transfection with pDNA alone (2 μg per well) was used as a negative control. The cells were incubated with transfection mixtures for 48 h and processed for further staining. Briefly, the cells were washed with 1 mL PBS, detached from plastic with 0,5 mL TripLE™ Express Enzyme, pelleted at 500 rcf, + 4°C for 7 min and resuspended in 25 μL FACS-buffer (2 mM EDTA in PBS). The resulting suspension was counted and stained with PE-conjugated anti-OX40L antibody (cat. no. ab95656, Abcam, Cambridge, MA, USA) or isotypical control antibodies (ab136585, Abcam, Cambridge, MA, USA) for 30 min on ice in the dark. After the staining, the cells were washed with FACS buffer 2 times and used for flow cytometric analysis. Cell suspensions were analyzed using BD FACSAria™ III (BD Biosciences, Franklin Lakes, NJ, USA). Twenty thousand events were collected for each sample. The acquired data were analyzed using the Flowing Software 2.5.1. (Mr. Perttu Terho, Turku Centre for Biotechnology, Turku, Finland). The debris and dead cells were excluded based on forward scatter and side scatter coordinates. Fluorescence intensity histograms for all the analyzed samples in PE channel (λexcitation = 561 nm, detection at 582/15 nm) were acquired.

### 2.5 Mice and animal studies

Female Balb/C (for CT26gfp and 4T1 cell lines) and C57BL/6 (for B16F0 cell line) mice were supplied from the Laboratory of Animal Breeding Facility (Branch of Shemyakin-Ovchinnikov Institute of Bioorganic Chemistry, Puschino, Moscow Region, Russia) and maintained in the animal facility of the IBCH RAS (Moscow, Russia). Animals had access to food and water *ad libitum*.

All the investigated tumor models were subcutaneously implanted to the corresponding mice strain. Briefly, cells were harvested via trypsinization, washed with PBS, and 1 × 10^5 (for CT26gfp cell line) or 5 × 10^5 (for B16F0, 4T1 cell lines) viable cells were injected s.c. into the right flank of the mice using a 1 ml insulin syringe. The antitumor efficacy of the treatment was estimated through tumor size measurement and animal survival monitoring. The tumor volume was used as a measure of tumor size, it was estimated according to the following equation: Tumor volume = tumor width × tumor length × tumor height × 0,52. Tumor width, length and height were measured with a caliper.

The treatment was started when tumor size reached 20–60 mm3 (day 0), mice received OX40L/PPT, pDNA or PBS intratumorally, half of the tumor volume (0,04 or 0,08 mcg/mm3 of tumor volume). The intratumoral injection of the drug was repeated twice on days 2 and 6 after the treatment start in the case of CT26gfp and B16F0 tumor models and on the days 2 and 4 in the case of 4T1 tumor model due to rapid tumor growth and generalization. Control group remained intact. In the experiment with ICB, mice were given 200 mcg of anti-PD-1 antibody i.p. (InVivoMAb anti-mouse PD-1 (CD279), Cat #BE0146, BioXCell, US) or 200 mcg of isotype control IgG2a antibody i.p. (InVivoMAb rat IgG2a isotype control, anti-trinitrophenol, Cat #BE0089, BioXCell, US) four times once in four days.

To evaluate OX40 receptor expression in CT26gfp tumors, mice were euthanized by cervical dislocation when the tumor size reached 100 mm^3^. For immune cell population analysis by flow cytometry, mice were euthanized by cervical dislocation, and the tumors were harvested at predetermined time points (1 day prior to treatment end, 4- and 7-days post-treatment). In all cases, excision was carefully made to separate the tumor tissue from the surrounding tissues.

Animals were euthanized when tumor size reached 2000 mm^3^ or when they became moribund with severe weight loss, or any other sign of critical condition.

All the animal experimental protocols were approved by the Animal Committee at the IBCH RAS and performed in accordance with all local guidelines and regulations.

### 2.6 Immune cell population analysis and OX40 receptor staining

#### 2.6.1 Tumor cell suspension preparation

Mouse tumor (100–200 mm3) was washed with PBS and placed into 5 ml of lysis solution (DMEM/F12 with 1% FCS, Antibiotic-Antimycotic solution and 1 mg/ml Collagenase D from Clostridium histolyticum (cat. no. 1088866001, Roche, Germany)) and chopped into 2–3 mm3 pieces using scissors. The resulting pre-suspension was incubated at 37C in a CO2 incubator for 2 hours, transferred into a 15 ml tube, and intensively pipetted to obtain a homogeneous suspension.

The resulting suspension was filtered through a 40 µm Cell Strainer (cat. no. 93040, SPL LifeSciences) and centrifuged at 500 g for 7 min at 4˚C. The cell pellet was resuspended in 1 ml Red Blood Cell Lysing Buffer Hybri-Max (cat. no. R7757, Sigma, United Kingdom) and incubated for 1 min at room temperature (18–25˚C). Then, 14.5 ml of PBS were added to the cell suspension in Hybri-Max buffer and the resulting suspension was centrifuged at 500 g for 7 min at 4˚C. The cell pellet was resuspended in 3–5 ml of PBS and the cell suspension was counted and stored on ice until further antibody staining.

#### 2.6.2 Tumor cell suspension staining

The tumor cell suspension was first incubated with blocking anti-mouse CD16/CD32 antibodies (Mouse BD Fc Block™) for 10 minutes at room temperature in order to exclude non-specific cell staining. After that, the suspension was divided into 1*10^6 cell aliquots and stained with corresponding antibody panel (for 30 min, to identify immune cell populations) and DAPI (for 20 min, to discriminate dead cells, Bio-Rad) on ice in the dark. All the panels included conjugated antibodies against the pan-leukocyte marker CD45. The T cell panel included antibodies against CD3, CD4 and CD8, the NK cell panel - against CD3, CD11b, NK1.1 or CD49b (depending on the tumor model) and the myeloid cell panel - against F4/80, CD11b and CD11c. The panel for evaluation of the OX40 receptor, expression included antibodies against OX40 (cat. no. 350004, BioLegend, San Diego, CA, USA), CD3, and CD8. All the specifics regarding antibodies, such as catalogue numbers, vendors and dilutions, are listed in the **Table 1**. In addition, fluorescence minus one (FMO) controls were prepared to accurately identify gates with target cell populations. After the staining, cells were washed twice with FACS buffer (PBS, 2mM EDTA, 4˚C, 500g, 7 min) and filtered through a 40 µm Cell Strainer. Cell population analysis was performed using BD FACSAria III (BD Biosciences) and BD FACSDiva (BD Biosciences) software. The parameters of the FACSAria III were first adjusted according to the sample staining and FMO-controls. Twenty thousand events were collected for each sample. The acquired data were analyzed using the Flowing Software 2.5.1. (Mr. Perttu Terho, Turku Centre for Biotechnology, Turku, Finland). The debris and dead cells were excluded based on forward scatter, side scatter coordinates and DAPI staining.

**Table 1:** Fluorescent antibodies used to identify immune cell populations.

Target	Dye	Dilution	Vendor	Catalogue number	Antibody panel
CD45	PerCP/Cy5.5	1:50	BioLegend	103132	NK, T and myeloid
CD4	AlexaFluor488	1:50	BioLegend	100423	T
CD8a	APC-Cy7	1:50	BioLegend	100713	T
CD3	APC	1:50	BioLegend	100236	NK, T
NK1.1	PE	1:50	BioLegend	156504	NK
CD11b	FITC	1:50	BioLegend	101217	NK, myeloid
CD11c	APC-Cy7	1:50	BioLegend	117324	Myeloid
F4/80	PE	1:50	BioLegend	123110	Myeloid
CD49b	FITC	1:50	BioLegend	108905	NK

### 2.7 Statistical analysis

In the case of immune cell population analysis, Student’s t-test (two-sample assuming unequal variances) was used to compare the number of corresponding cells between control and OX40L/PPT-treated mice at each timepoint. Differences were considered statistically significant at p (one-tail) < 0.05.

## 3 Results

### 3.1 The PEG-PEI-TAT copolymer neutralizes the negative charge of plasmid DNA and forms nano-sized complexes with it

**Figure 1:**
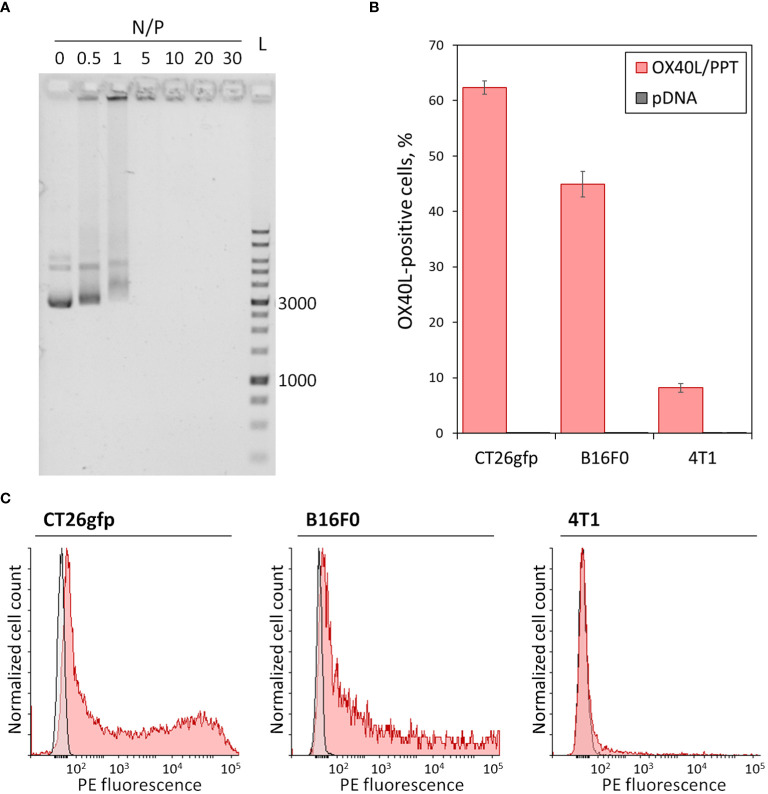
*In vitro* characterization of OX40L/PPT complexes. **(A)** – Gel mobility assay showing the neutralization of the negative charge of plasmid DNA. A total of 200 ng of plasmid DNA, Evrogen 1 Kb DNA Ladder (L), and complexes of PPT with OX40L-encoding plasmid DNA at various N/P charge ratios (0, 0.5, 1, 5, 10, 20 and 30) are shown. Values 1000 and 3000 correspond to 1 kbp and 3 kbp fragments of the ladder, respectively. **(B, C)** – Evaluation of OX40L/PPT transfection efficiency in several murine cancer cell lines via flow cytometry. CT26gfp, B16F0 and 4T1 murine cell lines were transfected with murine OX40L-encoding plasmid DNA alone (pDNA) or in a complex with PPT (OX40L/PPT and stained with PE-labeled anti-mouse OX40L monoclonal antibodies. **(B)** – The percentage of OX40L-positive cells upon transfection, mean over 3 replicates, error bars represent standard deviation (n = 3). **(C)** – Histograms representing the staining of CT26gfp, B16F0 and 4T1 cancer cell lines upon transfection with pDNA (grey) or OX40L/PPT (red).

Here we combined the previously described gene delivery system, PEG-PEI-TAT copolymer, PPT (23), with murine OX40L-encoding plasmid DNA (22). We further characterized the properties of OX40L/PPT complexes by performing DNA neutralization assay and measurement of size and zeta potential of the complexes. Since the negative charge of DNA prevents its free entry through the cell membrane into cells, its charge neutralization is crucial for effective gene delivery. We have previously shown the ability of PPT to condense pDNA and neutralize its negative charges at various N/P charge ratios. Here we confirmed the ability of PPT to neutralize the negative charge of DNA (**Figure 1A**). The effective diameter and polydispersity index of OX40L/PPT complexes were determined for N/P = 30 (was previously shown to be most effective in mice), and were 106.7 nm and 0.221, respectively, representing values applicable for cell transfection. The zeta-potential of the complexes was close to 0, which is consistent with the expected values, since PEG is used in the composition of the nanoparticles to form the hydrophilic corona around the PEI/DNA core, thereby providing close-to-zero zeta-potential of the whole complexes (23).

### 3.2 OX40L/PPT complexes transfect cancer cell lines of different origin resulting in OX40L expression

In order to confirm that OX40L/PPT complexes are capable of efficient cancer cell transfection, we have tested the *in vitro* transfection efficiency of the complexes using three murine cancer cell lines of different origin, CT26gfp (colon cancer), B16F0 (melanoma) and 4T1 (breast cancer). The cell lines were transfected with murine OX40L-encoding plasmid DNA alone (pDNA) or in a complex with PPT (OX40L/PPT) in full media. The transfection efficiency of each cell line was measured using flow cytometry, the percentage of OX40L-positive cells and fluorescence intensity were evaluated (**Figures 1B, C**).

The transfection of different cell lines with OX40L/PPT complexes resulted in significantly increased OX40L expression for all three used cell lines (**Figure 1B**) compared to transfection with plasmid DNA alone. Such an increase was observed both for percentage of OX40L-expressing cells (**Figure 1B**) and OX40L expression (measured indirectly as PE fluorescence intensity, **Figure 1C**). The transfection efficiency of OX40L/PPT in 4T1 cell line was lower than that in CT26gfp and 4T1 cell lines. Since the *in vitro* transfection efficiency does not necessarily correlate with the efficiency of further grafted tumor treatment, we further tested the efficiency of tumor treatment with OX40L/PPT in all three tumor models.

We have additionally evaluated the percentage of dead cells post-transfection with OX40L/PPT through staining of the cells with DAPI. We observed that OX40L/PPT complexes show modest toxicity toward B16F0 (but not CT26gfp or 4T1) cells (**Supplementary Table 1**), as in, the percentage of dead cells upon transfection with OX40L/PPT was greater than that with pDNA alone. However, the observed increase in cell death was moderate, and we found OX40L/PPT to be applicable for further *in vivo* experiments.

### 3.3 The effect of intratumoral OX40L/PPT administration on tumor growth and survival in multiple murine tumor models

**Figure 2:**
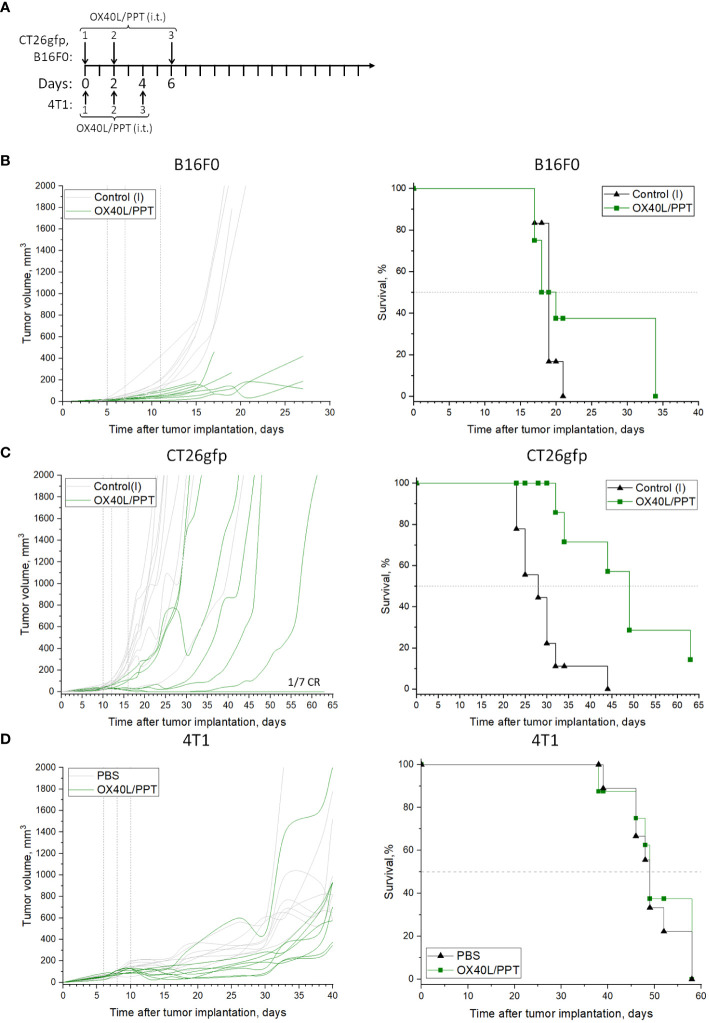
The effect of OX40L/PPT administration on tumor growth and survival. The treatment schemes are shown **(A)** for CT26gfp, B16F0 (top) or 4T1 (bottom) tumor models. Individual tumor growth (left) and Kaplan-Meier survival curves (right) are shown for mice inoculated with B16F0 (**B**, n ≥ 6), CT26gfp (**C**, n ≥ 7) and 4T1 (**D**, n ≥ 8) tumors. Mice were intratumorally treated with murine OX40L-encoding plasmid DNA in a complex with PPT (OX40L/PPT). As a control, the tumors were left intact (Control(I)), or treated with phosphate-buffered saline (PBS). Vertical lines on individual tumor growth plots indicate injection days. Horizontal line on survival plots indicates median survival. CR, complete response.

We next evaluated the effect of intratumoral OX40L/PPT administration on tumor growth and animal survival using the *in vitro* tested cell lines, CT26gfp, B16F0 and 4T1 (**Figure 2**).

First, we compared the effect of murine OX40L-encoding plasmid DNA alone (pDNA) or in a complex with PPT (OX40L/PPT) on subcutaneous B16F0 melanoma tumor growth. The treatment scheme is presented in **Figure 2A**. While pDNA alone barely affected tumor growth and animal survival (**Supplementary Figure 1**) in B16F0 tumor model, OX40L/PPT treatment resulted in significant tumor growth reduction (**Figure 2B**). While median survival did not differ between intact control and OX40L/PPT (**Figure 2B**), 3 animals (37.5%) in the OX40L/PPT group lived 13 days longer than the last animal in the control group (34 vs 21 days, respectively). Thus, OX40L/PPT treatment resulted in significant tumor growth reduction in B16F0 subcutaneous melanoma model, and showed a tendency to improve animal survival. Since we did not observe substantial effect of pDNA alone on tumor growth and animal survival compared to intact control, we have excluded this group from further experiments on CT26gfp and B16F0 to avoid unnecessary animal sacrifice and comply with the 3R principle for the conduct of research.

Then, we estimated the effect of OX40L/PPT on subcutaneous CT26gfp colon cancer tumor growth (**Figure 2C**, **Supplementary Figure 2**). OX40L/PPT was administered according to the same treatment scheme as in B16F0 tumor model (**Figure 2A**). Like in B16F0, OX40L/PPT treatment resulted in tumor growth reduction. Moreover, in CT26gfp tumor model OX40L/PPT treatment significantly improved animal survival (median survival changed from 28 to 49 days upon treatment) and resulted in 1 complete response. The responded mouse was then rechallenged and showed protective immunity against CT26gfp tumor growth (**Supplementary Figure 2**). Thus, OX40L/PPT treatment resulted in tumor growth reduction and significant survival enhancement in CT26 subcutaneous colon cancer model, and led to protective immunity against CT26gfp colon cancer cells.

Finally, we estimated the effect of OX40L/PPT on subcutaneous 4T1 breast cancer tumor growth (**Figure 2D**, **Supplementary Figure 3**) compared to injection of phosphate-buffered saline (PBS). The treatment scheme was modified due to rapid tumor growth (**Figure 2A**). While OX40L/PPT treatment initially showed a tendency to suppress tumor growth, this effect was not enough to enhance animal survival due to rapid tumor progression. This may be explained by low *in vitro* transfection efficiency of this particular cell line compared to CT26gfp or B16F0 (**Figures 1B, C**), or by the use of non-optimal treatment scheme.

Overall, we observed that OX40L/PPT treatment successfully inhibited tumor growth in B16F0 and CT26gfp tumor models and showed a tendency to inhibit 4T1 tumor growth, though the extent of this inhibition could be improved. No irAE signs, such as weight loss and cachexia, were observed in all 3 tumor models, which indicates good treatment tolerability in mice.

### 3.4 The effect of intratumoral OX40L/PPT administration on immune stromal cells

**Figure 3:**
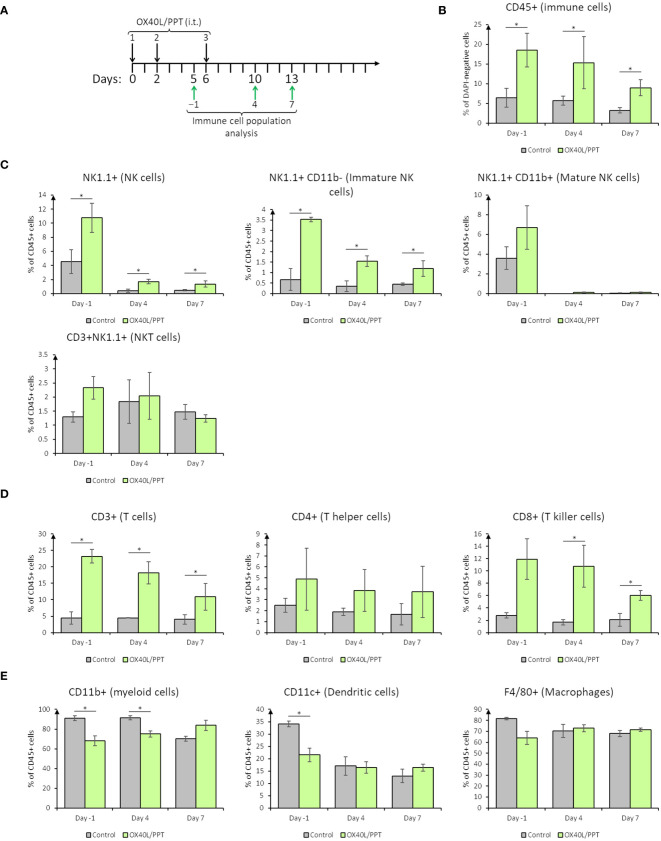
The effect of intratumoral nanoparticle administration on immune stromal cells in B16F0 tumor model. **(A)**, scheme of i.t. OX40L/PPT administration and immune cell population analysis timing; **(B)**, overall tumor immune cell infiltration estimation; **(C)**, stromal NK-cell populations analysis; **(D)**, stromal T-cell populations analysis; **(E)**, stromal myeloid cell populations analysis. Histograms showing the mean population % in DAPI-negative (alive, **B**) or CD45+ **(C–E)** cells are shown (n=3). Error bars represent SD. *, p < 0.05.

To confirm our hypothesis on immune stromal cell activation by OX40L/PPT, we have tested the immune response of B16F0 (**Figure 3**) and CT26gfp (**Supplementary Figure 4**) tumors at 3 different time points during the treatment course (days -1, 4 and 7 post-treatment, **Figure 3A**) by analyzing immune cell populations within the tumors. The analyzed populations included immune cells overall (CD45+), T cells (CD4+, CD8+), NK cells (including NKT cells, CD11b+/− NK cells), macrophages and dendritic cells.

In B16F0 tumor model, we have observed that overall tumor immune cell infiltration upon treatment with OX40L/PPT is greater than that in the control group on all the studied days (**Figure 3B**). The percentage of CD45-positive cells tends to be stably elevated during the treatment (day -1) and on day 4 post-treatment with OX40L/PPT, while by the 7^th^ day post-treatment it tends to decrease (**Figure 3B**). This elevation of the percentage of immune cells may also be explained by the cancer cell death, which leads to the change in cancer/immune cell ratio. Therefore, two effects – tumor immune cell infiltration elevation and cancer cell death – may co-occur during treatment.

**Figure 4:**
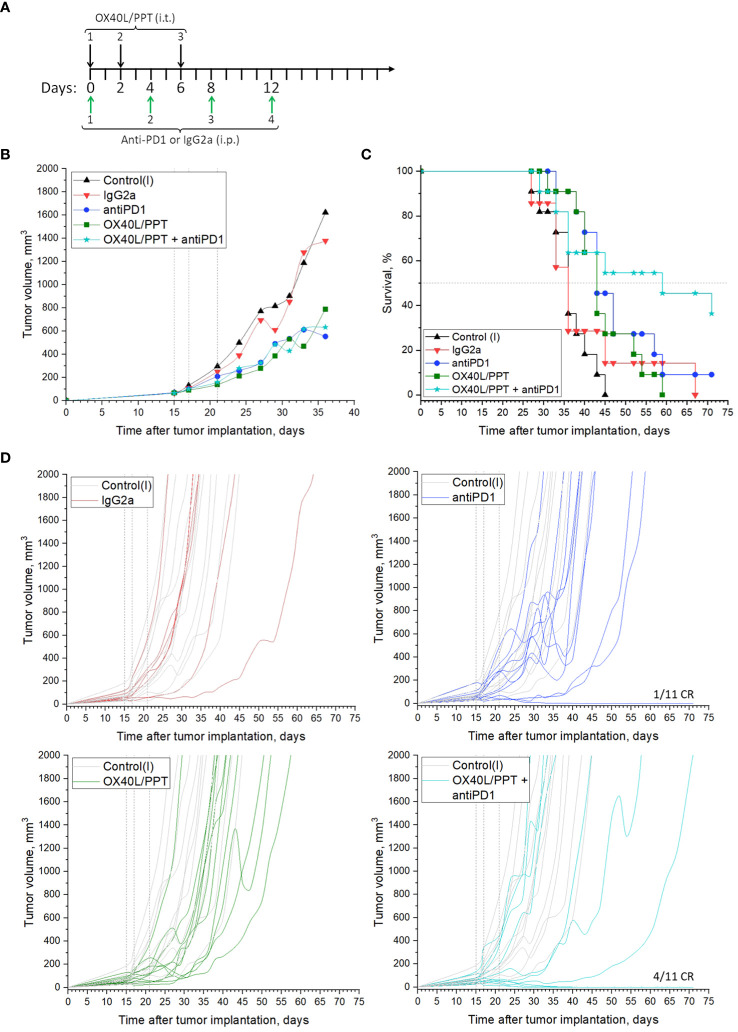
The effect of OX40L/PPT combination with PD-1 checkpoint blockade on CT26gfp tumor model. **(А)** – treatment scheme; **(B)** – mean tumor growth curves; **(C)** – Kaplan-Meier survival curves; **(D)** - individual tumor growth curves. The mice were untreated (Control (I), n = 11); treated with intraperitoneal (i.p.) control IgG2a antibodies (IgG2a, n = 7); anti-PD-1 antibodies i.p. (antiPD1, n = 11); intratumoral (i.t.) OX40L/PPT (OX40L/PPT, n = 11), or a combination of OX40L/PPT with anti-PD-1, (OX40L/PPT + antiPD1, n = 11). Vertical lines on individual tumor growth plots indicate OX40L/PPT injection days. Horizontal line on survival plots indicates median survival. CR, complete response.

Looking closer into specific immune cell populations, we have observed changes in percentage of both cells of innate and adaptive immunity. NK cell staining (**Figure 3C**) revealed that the percentage of NK cells (CD45 and NK1.1-positive) in B16F0 tumors increased upon treatment compared to control tumors (**Figure 3C**, top left). Interestingly, the main contributors into this increase were immature (25), that is, CD45 and NK1.1-positive, CD11b-negative NK cells, while the percentage of mature (25), that is, CD45, NK1.1 and CD11b-positive, NK cells and NKT (CD45, NK1.1 and CD3-positive) cells did not change in the treated tumors compared to control (**Figure 4C**). T cell staining (**Figure 3D**) also revealed that the percentage of T cells (CD45 and CD3-positive) in B16F0 tumors increased upon treatment compared to control tumors (**Figure 3D**, left), with that percentage slowly decreasing from day -1 to day 7 post-treatment. In this case, the main contributors into this increase were CD8-positive T killer cells, indicating active antitumoral immune response occurring upon treatment (**Figure 3D**, center). In turn, the percentage of CD4-positive T helper cells did not change compared to control (**Figure 3D**, right). Stromal myeloid cells staining (**Figure 3E**) revealed, that CD11b-positive myeloid cells comprise most of the immune cell population in control tumors, and this percentage decreases upon treatment with OX40L/PPT (**Figure 3E**, left, days -1 and 4). Also, the percentage of dendritic cells (CD45 and CD11c-positive) decreased during the treatment (day -1), but this decrease was not observed after the treatment end (days 4 and 7, **Figure 3E**, middle). No difference in the percentage of intratumoral macrophages (CD45 and F4/80-positive) was detected (**Figure 3E**, right).

Overall, in B16F0 tumor model we observed significant changes in intratumoral immune cell populations – while the percentage of antitumoral effector NK and T killer cells increased, the percentage of pro-tumoral myeloid cells decreased upon OX40L/PPT treatment.

In the case of CT26gfp tumors, a significant difference in intratumoral immune cell populations was detected only for dendritic cells on days 4 and 7 post-treatment (**Supplementary Figure 4**). Interestingly, the percentage of dendritic cells first decreased at day 4, and then increased at day 7 post-treatment in the tumors treated with OX40L/PPT compared to untreated tumors. Though B16F0 showed more significant results in immune cell population analysis, we might have not detected significant changes in CT26gfp-treated tumors due to estimation of percentages of immune cell population, not their activation state or marker expression level.

We have additionally estimated the OX40 receptor expression in CT26gfp tumors (**Supplementary Figure 5**). While cancer cells showed no expression of OX40, the surface OX40 protein was detected in 7.5% of immune cells, 19.4% of T cells, 9.3% of cytotoxic T cells (CD8+), and 4.5% of non-T leukocytes. Overall, the receptor was quite abundant in the immune cells of tumor stroma, indicating the possibility of its activation with OX40L.

### 3.5 The therapeutic efficacy of OX40L/PPT nanoparticles is significantly improved upon combination with immune checkpoint blockade

As mentioned above, we observed that OX40L/PPT treatment successfully inhibited tumor growth, though the extent of this inhibition could be improved. Moreover, the number of intratumoral T-killer and NK cells increased upon OX40L/PPT treatment. Therefore, an obvious improvement strategy would be to combine intratumoral OX40L/PPT with systemic checkpoint inhibition, since OX40L/PPT-induced antitumoral T-cell-mediated immune response might be enhanced by the checkpoint blockade inhibition. Here, we chose to combine OX40L/PPT treatment with systemic inhibition of PD-1 checkpoint.

We chose to evaluate the effect of OX40L/PPT combination with ICB on CT26gfp tumor model for several reasons. First, the use of OX40L/PPT on B16F0 cell line *in vitro* showed modest toxicity toward the cancer cells (**Supplementary Table 1**), and we aimed to evaluate the effect of OX40L expression itself, not the effect of additional OX40L/PPT toxicity. Besides, the observed antitumoral effect in CT26gfp model was more pronounced than that in B16F0, therefore we chose to evaluate the combination therapy in CT26gfp model. Though B16F0 tumors showed more significant immune population changes, these results did not discourage us from evaluating the combination efficiency in CT26gfp, since in immune cell population analysis we evaluated only immune cell population percentages, not their activation state or marker expression level, therefore we might have not detected significant changes in CT26gfp-treated tumors.

We estimated the effect of combination of OX40L/PPT with anti-PD-1 monoclonal antibodies on subcutaneous CT26gfp colon cancer tumor growth (**Figure 4**, **Supplementary Figure 5**). The treatment scheme is shown in **Figure 4A**. Briefly, mice were treated with OX40L/PPT i.t. as described above, anti-PD-1 antibodies i.p. (4 injections once every 4 days), combination of OX40L/PPT with anti-PD-1, control IgG2a antibodies i.p. (4 injections once every 4 days), or untreated (Control (I)).

As predicted, the combination of OX40L/PPT with anti-PD-1 significantly improved the efficiency of OX40L/PPT therapy (**Figures 4B–D**). While mean tumor growth did not differ between experimental groups (OX40L/PPT, anti-PD-1, OX40L/PPT + anti-PD-1, **Figure 4B**), individual tumor growth was improved significantly upon combination compared to both OX40L/PPT and anti-PD-1 (**Figure 4D**). We observed 0/11 (OX40L/PPT) and 1/11 (anti-PD-1) complete responses (CR) in single-agent treatment groups, and 4/11 complete responses in OX40L/PPT + anti-PD-1 group. Moreover, the animal survival was significantly enhanced in the combination group compared to single agent groups – while median survival in both OX40L/PPT and anti-PD-1 was 43 days, it increased to 59 days in the OX40L/PPT + anti-PD-1 group (**Figure 4C**). All the completely responded mice were then rechallenged and showed protective immunity against CT26gfp tumor growth (**Supplementary Figure 6**). No irAE signs, such as weight loss and cachexia, were observed. Thus, the combination of OX40L/PPT with PD-1 immune checkpoint blockade resulted in significant treatment efficiency enhancement in CT26gfp subcutaneous colon cancer model, and led to protective immunity against CT26gfp colon cancer cells.

## 4 Discussion

Immunotherapy is one of the rapidly developing cancer therapy fields, which has recently changed the paradigm of cancer treatment (1). One of the immunotherapeutic approaches, the immune checkpoint blockade (ICB), provides complete tumor regression even at advanced metastatic stages. However, only about 30% of patients respond to this type of therapy (26). Another immunotherapeutic approach is the use of immunostimulatory proteins, such as recombinant cytokines (IL2, IL12, IL15, CXCL9, *etc. (* 12, 13)) or T cell co-stimulatory ligands (OX40L, 4–1BBL, *etc. (* 14)). A major disadvantage of currently approved immunotherapeutics is the systemic administration, which leads to severe (grade ≥ 3) systemic immune-related adverse events (irAEs), such as colitis, endocrinopathy, nephritis, liver toxicity, rash, pruritus or pneumonitis (16, 27). Meanwhile the local intratumoral administration of immunotherapeutics as proteins (recombinant proteins, monoclonal antibodies) does not usually lead to pronounced antitumoral effects due to short protein half-life (hours) (28). One way to overcome these disadvantages is intratumoral gene-immune therapy, which allows for prolonged (days) and localized protein expression (29). All the currently approved anticancer gene therapeutics (i.e. talimogene laheprovec, IMLYGIC (30)) are administered intratumorally, which allows to increase the drug safety through the drug action localization and reduction of off-target effects. Gene therapeutics are particularly suitable for the expression localization, since in this case the localization is double-step (step 1, intratumoral injection; step 2, the need for protein to be synthesized via cellular protein biosynthesis machinery).

Since nucleic acids (NA) alone are not usually capable of intracellular entry due to the negative charge of phosphate backbone, and may be unstable and degraded in the extracellular matrix, a way to provide the stability and the ability to enter the cells is necessary for gene therapy. The most common approaches to provide these abilities are the use of viruses, liposomes and cationic polymers to envelop the nucleic acids. Each of the listed nucleic acid carrier types has its own advantages and disadvantages, which are regularly and extensively reviewed (30–33). In this study, we use the previously described PEG-PEI-TAT cationic copolymer, PPT (23), as the nucleic acid carrier. Briefly, PEI is used as the main cationic component, which allows for the negative charge neutralization; PEG is used to reduce the unnecessary interaction of the complexes with proteins by forming the hydrophilic corona around the PEI/NA core, and TAT-peptide is used to enhance the cell- and nucleus-penetrating abilities of the resulting complexes (23). Moreover, the use of PPT in a complex with plasmid DNA bearing therapeutic genes led to target protein expression and pronounced antitumoral effect in several murine tumor models (20).

Several types of nucleic acids are used in non-viral gene therapeutics, such as plasmid DNA, mRNA and siRNA (34). In this study, we chose to use the plasmid DNA due to its higher stability, higher capacity and lower production costs compared to the other types of nucleic acids. As the immunotherapeutic component, we chose to use the T cell co-stimulator ligand OX40L, since T-cell co-stimulatory molecules can be used both as monotherapy and in combination with ICB, and targeting the OX40 receptor as antitumor treatment was demonstrated to be effective in numerous preclinical and clinical trials (extensively reviewed by Yadav and Redmond (15)). The OX40 receptor is present on T, NK, NKT and several other types of cells, and the antitumoral effect of its stimulation is mainly attributed to activation, sustainment and proliferation of the effector immune cells via NF-kB and PI3K-PKB (protein kinase B/Akt) signaling (15). The use of natural OX40 ligand is particularly attractive due to lower immunogenic potential compared to antibodies and other similar types of proteins.

Various agonists of OX40 receptor are currently actively investigated in clinical trials. The types of agonists include anti-OX40 monoclonal antibodies (i.e, murine anti-human OX40 agonist MEDI6469 and its humanized version MEDI0562 (35)), bispecific antibodies (i.e., a human IgG1 bispecific mAb ATOR-1015 that targets both OX40 and CTLA-4 (36) or a bispecific antibody FS120 targeting OX40 and 4–1BB (37)), adenoviruses (i.e., OX40L-encoding oncolytic adenovirus DNX-2440 (38)), and mRNA-based therapeutics (i.e., Moderna’s mRNA-2416 (39) or mRNA-2752 (40)). Of these, only gene therapeutics, DNX-2440, mRNA-2416, and mRNA-2752 are administered intratumorally, while the systemically-administered therapeutics yield systemic toxicity. Moreover, DNX-2440 is adenovirus-based, which may lead to limitations in the practical use, such as immunogenicity, cytotoxicity, potential carcinogenicity and production cost.

The obtained OX40L/PPT complexes showed full neutralization of the negative charge of plasmid DNA (**Figure 1A**). The effective diameter of the complexes showed values applicable for *in vitro* cell transfection. We further tested the *in vitro* transfection efficiency of the complexes using 3 murine cancer cell lines of different origin, CT26gfp (colon cancer), B16F0 (melanoma) and 4T1 (breast cancer). These cell lines were chosen due to ability to form subcutaneous syngeneic tumors upon grafting to mice. We also used CT26gfp cell line instead of CT26.WT to be able to further estimate intratumoral immune cell population dynamics. The transfection efficiency was estimated with help of fluorescent anti-OX40L (PE) staining of the cells transfected with OX40L-encoding plasmid DNA alone or OX40L/PPT complexes and further flow cytometry. In all 3 cases, the percentage of OX40L-positive cells in OX40L/PPT group was significantly greater than that of pDNA group (**Figure 1B**), indicating that OX40L/PPT complexes are capable of effective cancer cell transfection. Though the *in vitro* transfection efficiency does not necessarily correlate with the efficiency of further grafted tumor treatment, the obtained result encouraged us to further test the efficiency of tumor treatment with OX40L/PPT. We did not evaluate the *in vivo* OX40L expression during the therapy course, since we used native murine OX40L gene. This limitation doesn’t allow us to confidently attribute the elevation in intratumoral OX40L protein to the administered therapy due to possible endogenous expression of the protein in the studied murine tumor models. In example, such elevation could be due to the reaction of murine immune cells to therapy, not to the administration of gene itself, or the natural growth of the tumor. It is not possible to rule out these factors using controls, since the background expression level of the protein would be different for each mouse.

Due to the observed *in vitro* results, we have tested the efficiency of the OX40L/PPT therapy in B16F0, CT26gfp and 4T1 subcutaneously grafted tumor models (**Figure 2**). OX40L/PPT was injected three times (**Figure 2A**). As a control, we used either untreated intact tumors (B16F0, CT26gfp), or tumors treated with PBS (4T1). The treatment efficiency of OX40L-encoding plasmid DNA alone was also tested in one of the 3 tumor models (B16F0, **Supplementary Figure 1**). The treatment with OX40L/PPT had an effect on tumors compared to controls in all 3 cases, though, interestingly, the observed effect varied between the tumor models. No irAE signs, such as weight loss and cachexia, were observed in all 3 tumor models.

In case of B16F0, we observed a significant reduction in mean tumor growth, however, the median animal survival did not differ between treated with OX40L/PPT group and control groups (intact control and OX40L-encoding plasmid DNA alone, **Figure 2B**, **Supplementary Figure 1**). Despite this, we observed that for several animals (37.5% of the group) the survival was prolonged for almost 2 weeks upon treatment, indicating that a certain cohort of animals responds better to OX40L/PPT treatment. Interestingly, B16F0 tumors showed much more pronounced immune response compared to CT26gfp (**Figure 3**, **Supplementary Figure 4**). Moreover, the immune response in these tumors was strongly shifted towards effector phenotype (CD8+ T cell and NK cell proportions among the immune cells of tumor microenvironment increased significantly throughout the course of the treatment compared to untreated control). Since no irAE signs were observed upon treatment, apparently, the treatment regimen should be modified for more effective response, or OX40L/PPT treatment should be combined with other immunostimulators, or systemic immune checkpoint blockade (ICB) in order to achieve more pronounced effect in this tumor model. Moreover, we compared only 3 animals per timepoint between control and treated groups in the immune response analysis, therefore, the observed result might not correctly represent the overall population response to the drug.

In CT26gfp tumor model, we observed both the reduction in mean tumor growth and the increase in animal survival. The effect of OX40L/PPT treatment on animal survival was very pronounced – the median survival increased almost twice compared to control, and one complete response (CR) was observed (**Figure 2C**). The completely responded mouse was further rechallenged with CT26gfp tumor cells and showed protective immunity against this type of tumor cells (**Supplementary Figure 2**). Despite the great effect on animal survival, no differences were revealed between treated with OX40L/PPT and untreated tumors during the analysis of intratumoral immune cell populations (**Supplementary Figure 4**). This may be due to individual tumor composition variability, small sample size, and the fact that the effect of OX40L/PPT on the growth of this type of tumors is caused by the change in activated immune cell populations, and not the proportion of these immune cell populations among all intratumoral immune cells (the individual immune cell type activation was not evaluated in this study due to the method limitations).

In 4T1 tumor model, we observed initial tumor growth inhibition upon OX40L administration compared to control (PBS administration), however, this inhibition stopped when the treatment course was finished, and in the end both animal survival and tumor growth did not differ compared to control group due to rapid tumor generalization (**Figure 2D**).

Overall, we observed that OX40L/PPT treatment successfully inhibited tumor growth in B16F0 and CT26gfp tumor models and showed a tendency to inhibit 4T1 tumor growth, though the extent of this inhibition could be improved. We find the observed tendency for 4T1 tumor growth inhibition encouraging, since this model is rapidly-growing and treatment-resistant, and had lower *in vitro* transfection efficiency with PPT than that of B16F0 and CT26gfp (**Figures 1B, C**), which might explain the observed treatment efficiency limitation. The strategies for the improvement of the efficiency of OX40L/PPT treatment include the optimization of treatment scheme, the combination of OX40L/PPT with other genetically-encoded immunostimulators (i.e., 4–1BBL, CD80/86, cytokines), and the combination of OX40L/PPT with ICB. Due to the observed increase in the number of intratumoral T-killer and NK cells upon OX40L/PPT treatment, the combination of OX40L/PPT with ICB seems to be the most promising approach.

Finally, we estimated the effect of combination of OX40L/PPT with anti-PD-1 ICB on CT26gfp tumor growth. We observed that the effect on the tumor growth of OX40L/PPT alone, anti-PD-1 alone or their combination did not differ between the three groups, though it was obviously pronounced compared to control groups (control systemic antibody, IgG2a, and intact control), **Figure 4**. However, the combination of anti-PD-1 therapy with OX40L/PPT had a significant effect on animal survival enhancement – the median survival in the combination group increased by 16 days compared to the treatment with single OX40L/PPT or anti-PD-1. Moreover, we have observed 4/11 complete responses in the combination group, compared to 0/11 and 1/11 in single agent groups (**Figure 4**). All the completely responded mice were then rechallenged and showed protective immunity against CT26gfp tumor growth (**Supplementary Figure 6**). No irAE signs, such as weight loss and cachexia, were observed. Thus, the combination of OX40L/PPT with PD-1 immune checkpoint blockade resulted in significant treatment efficiency enhancement in CT26 subcutaneous colon cancer model, and led to protective immunity against CT26gfp colon cancer cells.

Initially we assumed that the use of CT26gfp cell line might provide additional immunogenicity due to the presence of EGFP. CT26.WT cell line is mostly considered moderately immunogenic (41), though several studies mention it as poorly (42) or even highly (43) immunogenic depending on the immunogenicity criteria. The treatment of CT26gfp tumors with anti-PD1 antibody led to 1/11 CR, which is in accordance with experiments of other researchers on CT26.WT tumors, i.e., Chaudhri et al. (44) observed 2/9 CRs upon PD1 ICB in s.c. CT26.WT tumor model. Therefore, we assume that the additional immunogenicity that could be provided by the presence of EGFP was insignificant.

Thus, we observed that OX40L/PPT treatment had a significant effect on tumor growth and animal survival in several murine tumor models. This effect was also detected at the intratumoral immune cell populations level in the case of B16F0 tumor model. Moreover, OX40L/PPT treatment conferred protective immunity against CT26gfp tumor cells. However, the antitumoral effect of OX40L/PPT was not enough to enhance animal survival in 4T1 breast cancer tumor model. The strategies to enhance the antitumoral effect of OX40L/PPT gene-immune therapy may include (1) combination with ICB and (2) combination with other types of anticancer immunostimulatory molecules, such as chemokines, interleukins, or other T-cell co-stimulators.

## 5 Conclusion

In this study, we combined the previously described gene delivery system with OX40L-encoding plasmid DNA. The resulting OX40L/PPT combination led to self-assembly of nanoparticles, which were able to provide effective OX40L expression in several types of cancer cells. The *in vivo* antitumoral OX40L/PPT efficiency against several murine tumor models was also evaluated. Additionally, we investigated the effect of OX40L/PPT combination with anti-PD-1 ICB. OX40L/PPT nanoparticles showed a promising antitumor efficiency both as monotherapy and in combination with ICB. In B16F0 tumor model, OX40L/PPT treatment led to the increase in antitumoral effector NK and T killer cells and to the decrease in pro-tumoral myeloid cells populations within tumor stroma. The observed antitumoral effect of OX40L/PPT thus may be explained through intratumoral T and NK cell stimulation via OX40L.

## Data availability statement

The original contributions presented in the study are included in the article/**Supplementary Material**. Further inquiries can be directed to the corresponding author.

## Ethics statement

The animal study was approved by Animal Committee, Shemyakin-Ovchinnikov Institute of Bioorganic Chemistry RAS, Moscow, Russia. The study was conducted in accordance with the local legislation and institutional requirements.

## Author contributions

OR: Investigation, Visualization, Writing – original draft, Writing – review & editing, Validation. AK: Conceptualization, Investigation, Project administration, Supervision, Visualization, Writing – original draft, Writing – review & editing. OB: Conceptualization, Investigation, Project administration, Supervision, Writing – original draft, Writing – review & editing. SK: Investigation, Visualization, Writing – original draft, Writing – review & editing. VP: Conceptualization, Investigation, Project administration, Supervision, Writing – original draft, Writing – review & editing. MZ: Conceptualization, Investigation, Project administration, Supervision, Writing – original draft, Writing – review & editing. DD: Conceptualization, Investigation, Project administration, Supervision, Writing – original draft, Writing – review & editing. AS: Investigation, Writing – original draft, Writing – review & editing. ES: Investigation, Writing – original draft, Writing – review & editing. MBK: Investigation, Writing – original draft, Writing – review & editing. MOK: Investigation, Writing – original draft, Writing – review & editing. IA: Conceptualization, Investigation, Project administration, Supervision, Writing – original draft, Writing – review & editing.
