# Supplementary material for: Non-viral-mediated gene transfer of OX40 ligand for tumor immunotherapy
Source: Front Immunol. 2024 Jun 26;15:1410564. doi: 10.3389/fimmu.2024.1410564 (PMC11245119; doi:10.3389/fimmu.2024.1410564)
Supplement: Supplementary file 1 [file DataSheet_1.pdf]

# *Supplementary Material*

# *Non-viral-mediated gene transfer of OX40 ligand for tumor immunotherapy*

Olga A Rakitina[1*], Alexey I Kuzmich[1], Olga A Bezborodova[1], Sofia A Kondratieva[1], Victor V Pleshkan[1], Marina V Zinovyeva[1], Dmitry A Didych[1], Aleksandr V Sass[1], Eugene V Snezhkov[1], Maria B Kostina[1], Maksim O Koksharov[1,2] and Irina V Alekseenko[1,2]

[1]Shemyakin-Ovchinnikov Institute of Bioorganic Chemistry of the Russian Academy of Sciences, Moscow, Russian Federation

[2]Stagen LLC, Moscow, Russian Federation

*Corresponding author

Shemyakin-Ovchinnikov Institute of Bioorganic Chemistry of the Russian Academy of Sciences,

Miklukho-Maklaya st. 16/10, 117997, Moscow, Russian Federation

Tel.: +7 (915) 358-76-51

E-mail: rakitinaolga97@gmail.com

## 1 Table of contents:

Supplementary Figure 1. The effect of OX40L/PPT administration on B16F0 melanoma tumor growth and animal survival.

Supplementary Figure 2. The effect of OX40L/PPT administration on CT26gfp colon adenocarcinoma tumor growth and development of protective immunity against CT26gfp tumor cells.

Supplementary Figure 3. The effect of OX40L/PPT administration on 4T1 breast cancer tumor growth.

Supplementary Figure 4. The effect of intratumoral nanoparticle administration on immune stromal cells in CT26gfp tumor model.

Supplementary Figure 5. Profiling of OX40 receptor expression in CT26gfp tumors.

Supplementary Figure 6. Development of protective immunity against CT26gfp tumor cells upon combination of OX40L/PPT with PD1 checkpoint blockade.

Supplementary Table 1. Evaluation of OX40L/PPT transfection efficiency and cell death in several murine cancer cell lines via flow cytometry

## 2 Supplementary Figures

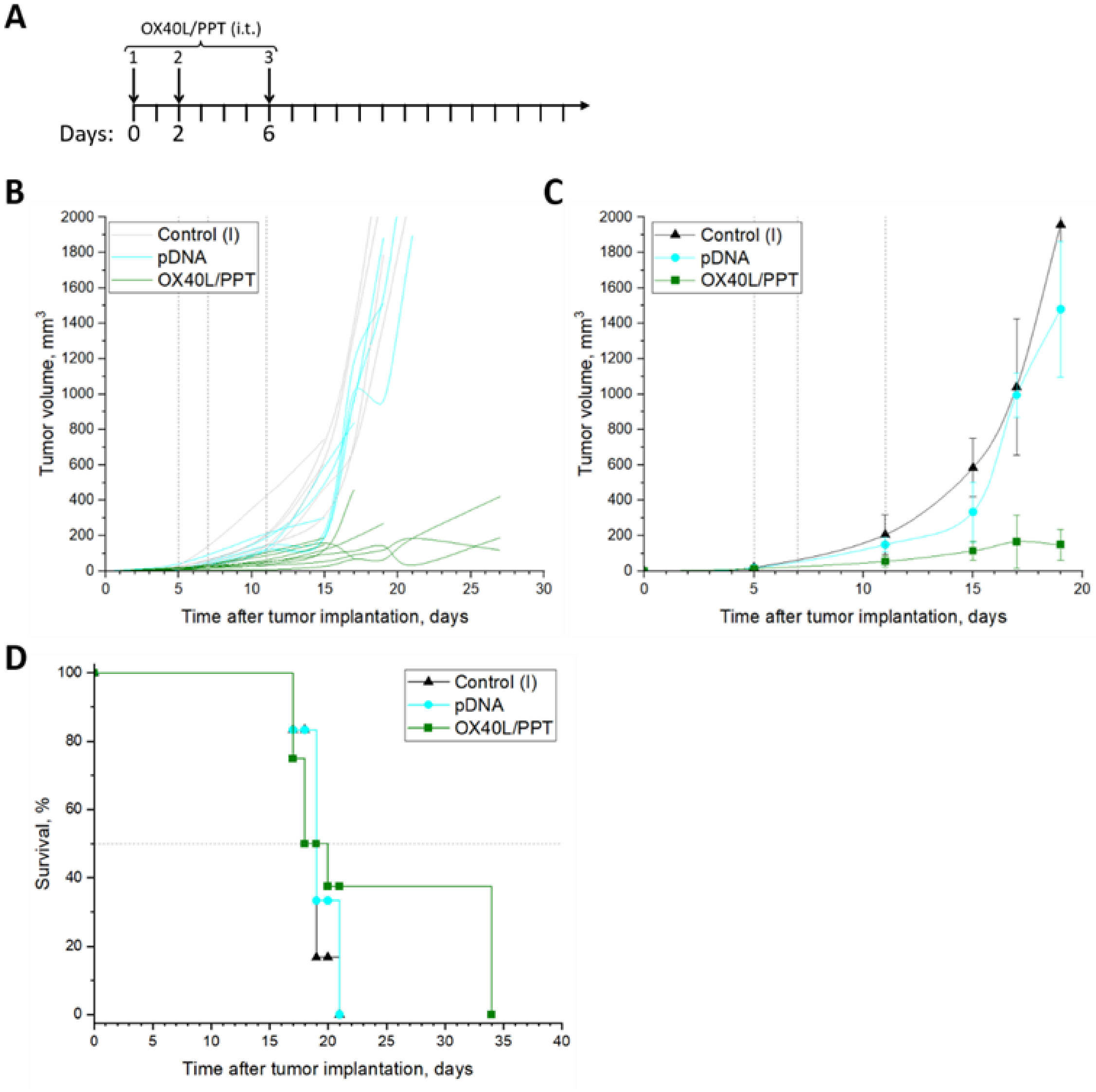


**Supplementary Figure 1.** The effect of OX40L/PPT administration on B16F0 melanoma tumor growth and animal survival. The treatment scheme is shown (A). Individual (B), mean (C) tumor growth and Kaplan-Meier survival curves (D) are shown, n ≥ 6. Mice were intratumorally treated with murine OX40L-encoding plasmid DNA alone (pDNA) or in a complex with PPT (OX40L/PPT). As a control, the tumors were left intact (Control(I)). Vertical lines on tumor growth plots indicate injection days. Horizontal line on survival plots indicates median survival.

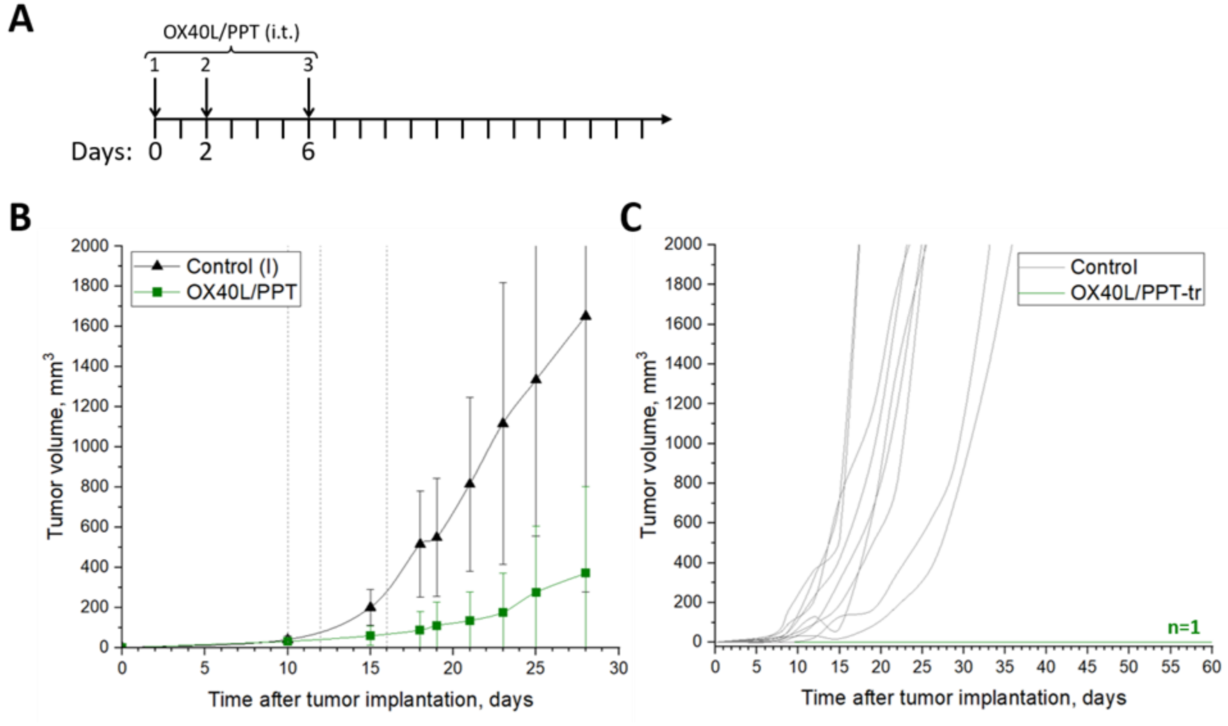


**Supplementary Figure 2.** The effect of OX40L/PPT administration on CT26gfp colon adenocarcinoma tumor growth and development of protective immunity against CT26gfp tumor cells. The treatment scheme (A) and mean tumor growth plots (B) are shown, n ≥ 7. In B, mice were intratumorally treated with murine OX40L-encoding plasmid DNA in a complex with PPT (OX40L/PPT). As a control, the tumors were left intact (Control(I)). Vertical lines on tumor growth plot indicate injection days. C, Individual tumor growth plots for OX40L/PPT-treated mice re-challenged with 100 000 CT26gfp tumor cells (n=1) or age-matched control mice (n = 9).

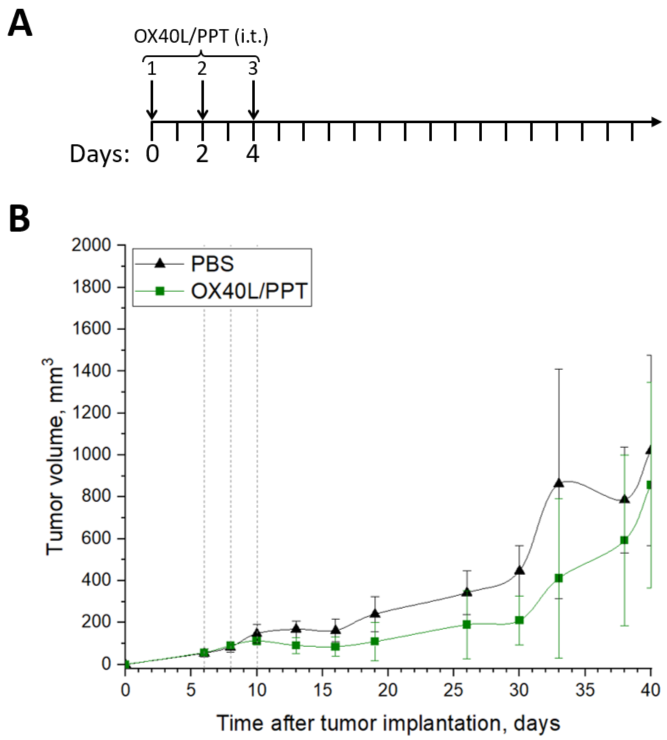


**Supplementary Figure 3.** The effect of OX40L/PPT administration on 4T1 breast cancer tumor growth. The treatment scheme (A) and mean tumor growth plots (B) are shown, n ≥ 8. Mice were intratumorally treated with murine OX40L-encoding plasmid DNA in a complex with PPT (OX40L/PPT) or phosphate-buffered saline (PBS). Vertical lines on tumor growth plot indicate injection days.

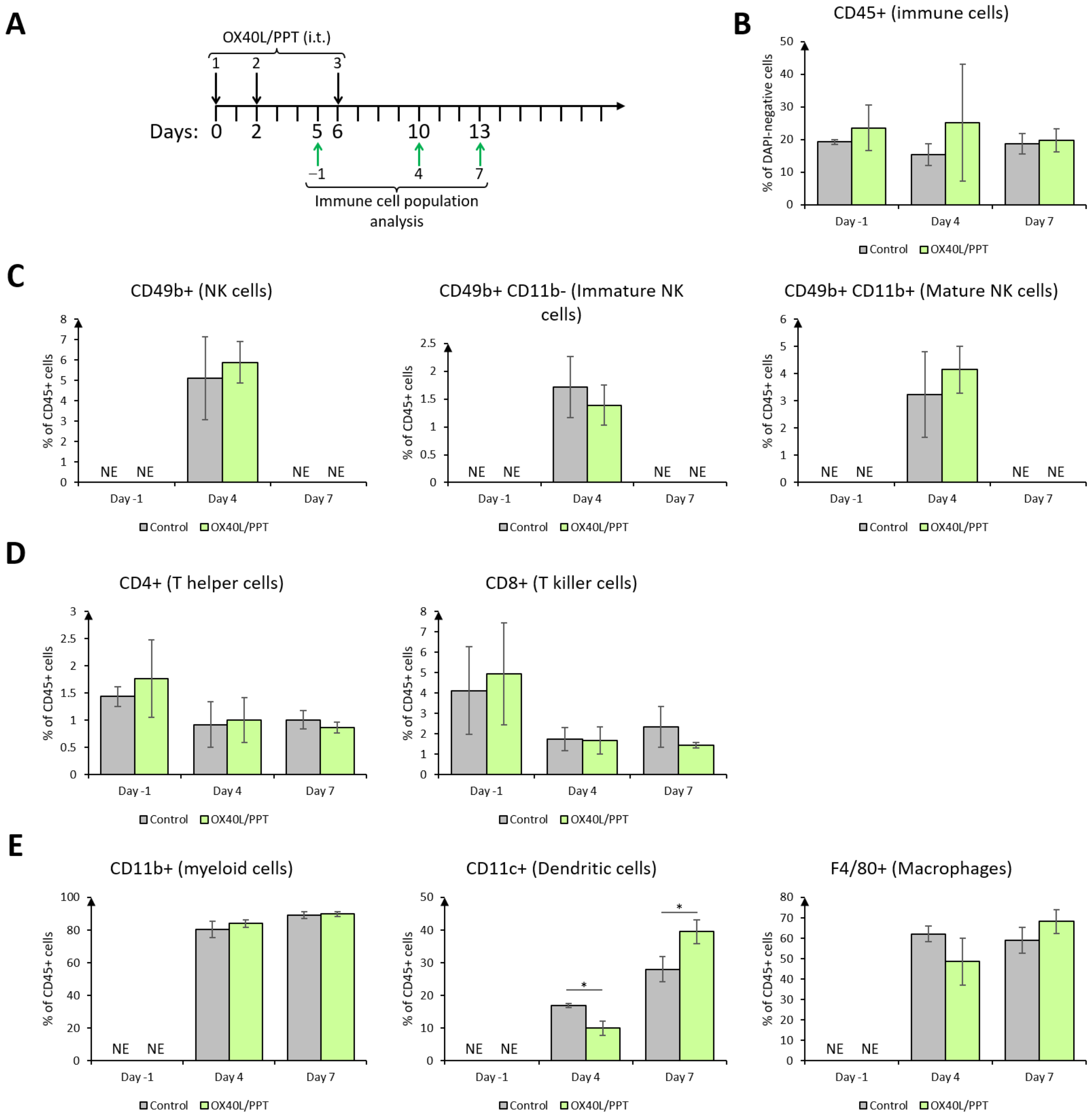


**Supplementary Figure 4.** The effect of intratumoral nanoparticle administration on immune stromal cells in CT26gfp tumor model. A, scheme of i.t. OX40L/PPT administration and immune cell population analysis timing; B, overall tumor immune cell infiltration estimation; C, stromal NK-cell populations analysis; D, stromal T-cell populations analysis; E, stromal myeloid cell populations analysis. Histograms showing the mean population % in DAPI-negative (alive, B) or CD45+ (C-E) cells are shown (n=3). Error bars represent SD. *, $p < 0.05$. NE – not estimated.

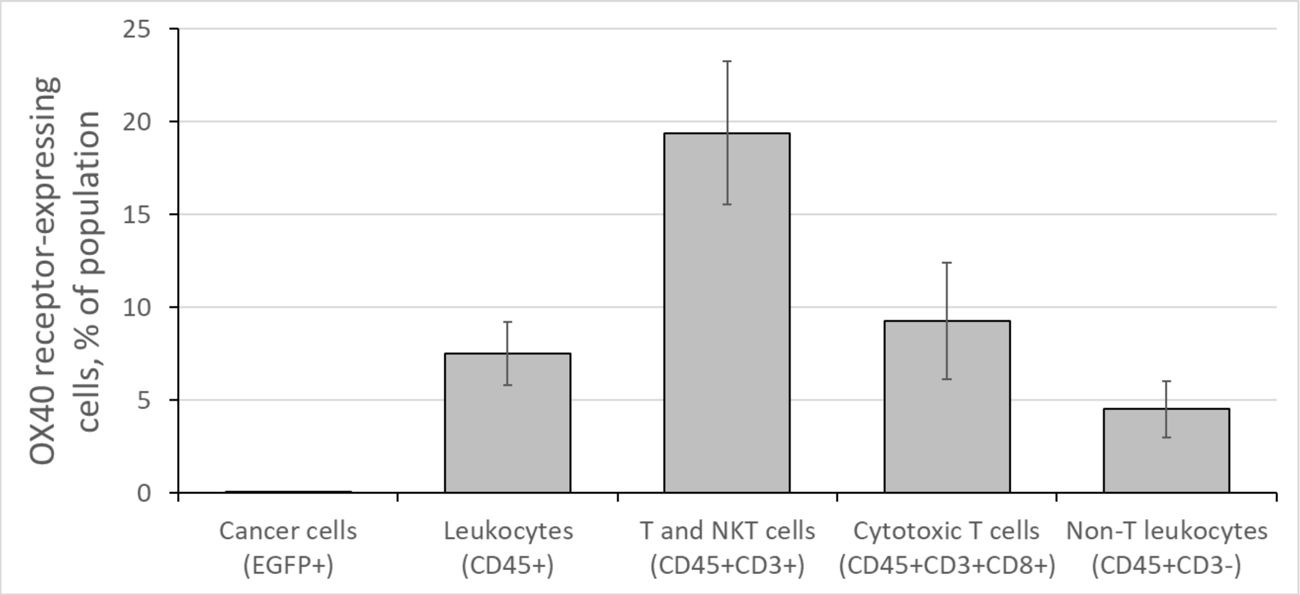


**Supplementary Figure 5.** Profiling of OX40 receptor expression in CT26gfp tumors. Histograms showing the mean percentage of OX40-positive cells in EGFP+ cancer cells, CD45+ leukocytes, CD45+ CD3+ T and NKT cells, CD45+ CD3+ CD8+ cytotoxic T cells and CD45 CD3- Non-T leukocytes are shown (n+ =3). Error bars represent SD.

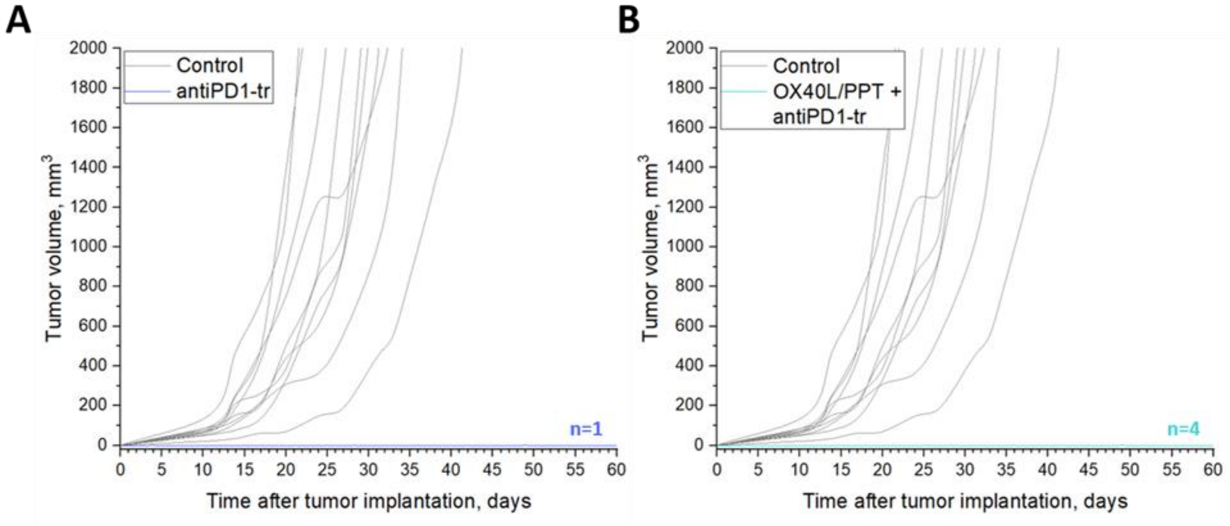


**Supplementary Figure 6.** Development of protective immunity against CT26gfp tumor cells upon combination of OX40L/PPT with PD1 checkpoint blockade. The individual tumor growth plots for anti-PD-1 (A, antiPD1-tr) or OX40L/PPT+anti-PD-1 (B, OX40L/PPT+antiPD1-tr)-treated mice re-challenged with 100 000 CT26gfp tumor cells (n=1, A, n=4, B) or age-matched control mice (n = 10).

## 3 Supplementary Tables

**Supplementary Table 1.** Evaluation of OX40L/PPT transfection efficiency and cell death in several murine cancer cell lines via flow cytometry. CT26gfp, B16F0 and 4T1 murine cell lines were transfected with murine OX40L-encoding plasmid DNA alone (pDNA, n=3), in a complex with PPT (OX40L/PPT, n=3), or with Lipofectamine 2000 (LFA, n=1) and stained with PE-labeled anti-mouse OX40L monoclonal antibodies, or isotypical antibodies (OX40L/PPT Isotypical, n=1). The transfected cells were also stained with DAPI to evaluate the percentage of dead cells. The values are represented as mean ± standard deviation for OX40L/PPT and pDNA.

| Transfection agent | OX40L/PPT (n=3) | pDNA (n=3) | LFA (n=1) | OX40L/PPT (n=1) |
|---|---|---|---|---|
| Staining | anti-OX40L | anti-OX40L | anti-OX40L | Isotypical |
| OX40L-positive cells, % | | | | |
| CT26gfp | 62.34 ± 1.23 | 0.04 ± 0.02 | 24.74 | 0.10 |
| B16F0 | 44.92 ± 2.28 | 0.02 ± 0.01 | 68.92 | 0.19 |
| 4T1 | 8.20 ± 0.78 | 0.05 ± 0.01 | 24.31 | 0.22 |
| Dead cells (DAPI+), % | | | | |
| CT26gfp | 5.28 ± 0.56 | 6.16 ± 0.32 | 14.43 | 5.14 |
| B16F0 | 17.50 ± 2.63 | 7.83 ± 1.73 | 12.19 | 10.27 |
| 4T1 | 8.55 ± 3.20 | 9.77 ± 1.02 | 17.81 | 17.53 |
